# Remnant cholesterol, stronger than triglycerides, is associated with incident non-alcoholic fatty liver disease

**DOI:** 10.3389/fendo.2023.1098078

**Published:** 2023-05-05

**Authors:** Yiping Cheng, Qiang Zhang, Haizhen Li, Guangshuai Zhou, Ping Shi, Xu Zhang, Liying Guan, Fang Yan, Chao Xu

**Affiliations:** ^1^ Department of Endocrinology and Metabolism, Shandong Provincial Hospital Affiliated to Shandong First Medical University, Jinan, Shandong, China; ^2^ Department of Endocrinology and Metabolism, Shandong Provincial Hospital, Shandong University, Jinan, Shandong, China; ^3^ Department of Critical Care Medicine, Zibo Central Hospital, Zibo, Shandong, China; ^4^ Department of Endocrinology, Dongying City District People Hospital, Dongying, Shandong, China; ^5^ Department of Scientific Research and Cooperation, Zibo Central Hospital, Zibo, Shandong, China; ^6^ Department of Health Examination Center, Shandong Provincial Hospital Affiliated to Shandong First Medical University, Jinan, Shandong, China; ^7^ Department of Pain Management, Shandong Provincial Hospital, Shandong University, Jinan, Shandong, China

**Keywords:** Non-alcoholic fatty liver disease, Remnant cholesterol, Triglyceride, Fat metabolization, Longitudinal retrospective cohort study

## Abstract

**Introduction:**

Non-alcoholic fatty liver disease (NAFLD) is characterized by excess accumulation of triglycerides within the liver. However, whether the circulating levels of triglycerides and cholesterol transported in triglyceride-rich lipoproteins (remnant cholesterol, remnant-C) are related to the occurrence of NAFLD has not yet been studied. This study aims to assess the association of triglycerides and remnant-C with NAFLD in a Chinese cohort of middle aged and elderly individuals.

**Methods:**

All subjects in the current study are from the 13,876 individuals who recruited in the Shandong cohort of the REACTION study. We included 6,634 participants who had more than one visit during the study period with an average follow-up time of 43.34 months. The association between lipid concentrations and incident NAFLD were evaluated by unadjusted and adjusted Cox proportional hazard models. The potential confounders were adjusted in the models including age, sex, hip circumference (HC), body mass index (BMI), systolic blood pressure, diastolic blood pressure, fasting plasma glucose (FPG), diabetes status and cardiovascular disease (CVD) status.

**Results:**

In multivariable-adjusted Cox proportional hazard model analyses, triglycerides (hazard ratio[HR], 95% confidence interval [CI]:1.080,1.047-1.113;p<0.001), high-density lipoprotein cholesterol (HDL-C) (HR, 95% CI: 0.571,0.487-0.670; p<0.001), and remnant-C (HR, 95% CI: 1.143,1.052-1.242; p=0.002), but not total cholesterol (TC) or low-density lipoprotein cholesterol (LDL-C), were associated with incident NAFLD. Atherogenic dyslipidemia (triglycerides>1.69 mmol/L, HDL-C<1.03 mmol/L in men or<1.29 mmol/L in women) was also associated with NAFLD (HR, 95% CI: 1.343,1.177-1.533; p<0.001). Remnant-C levels were higher in females than in males and increased with increasing BMI and in participants with diabetes and CVD compared with those without diabetes or CVD. After adjusting for other factors in the Cox regression models, we found that serum levels of TG and remnant-C, but not TC or LDL-C, were associated with NAFLD outcomes in women group, non-cardiovascular disease status, non-diabetes status and middle BMI categories (24 to 28 kg/m2).

**Discussion:**

In the middle aged and elderly subset of the Chinese population, especially those who were women, non-CVD status, non-diabetes status and middle BMI status (24 to 28 kg/m2), levels of triglycerides and remnant-C, but not TC or LDL-C, were associated with NAFLD outcomes independent of other risk factors.

## 1 Introduction

Non-alcoholic fatty liver disease (NAFLD), characterized by excessive intrahepatic lipid accumulation, is the most prevalent chronic liver disease in the world (1). In addition to progression from simple steatosis to nonalcoholic steatohepatitis (NASH), cirrhosis and hepatocellular carcinoma, NAFLD patients have an increased risk for cardiovascular disease (CVD) morbidity and mortality. Despite the huge investment in drug development, there are still no effective therapies targeting NAFLD. Clearly, identification and elimination of the risk factors that promote NAFLD development and progression are essential and promising therapeutic strategy that can reduce the incidence of NAFLD.

Because intrahepatic lipid accumulation results from lipid metabolism abnormalities, it takes for granted that dyslipidemia can cause NAFLD. However, there are very few studies on the role of different types of dyslipidemia in the development of NAFLD, and the research conclusions are inconsistent. Sun et al. have found the elevation of low-density lipoprotein cholesterol (LDL-C) level within the normal range appears to make a significant contribution to an increased risk of developing NAFLD (2) and previous studies have demonstrated that patients with NAFLD have significantly increased of oxidized LDL-C levels (3, 4). A cross-sectional and hospital-based study in Alexandria was performed to verify that NAFLD in outpatient schoolchildren aged 6-18years was significantly associated with high triglycerides (TG) and low high-density lipoprotein cholesterol (HDL-C) (5). A population-based study has shown NAFLD in 6814 participants aged 45-84 years was associated with higher fasting TG, lower serum HDL-C but no difference in total cholesterol (TC) or LDL-C (6). However, a detailed description, whether the progression of time affects the association between components of dyslipidemia and NAFLD, has been lacked in adults over the past decades.

Remnant cholesterol (Remnant-C) is the residue produced by triglyceride-rich lipoproteins (TRLs) metabolism, that is, chylomicrons (CM) and very low-density lipoproteins (VLDL) are lipolyzed by lipoprotein lipase (LPL) to lose TG and produce metabolic residues rich in cholesterol esters (7). Recently, studies have shown that remnant-C is highly correlated with coronary heart disease (CHD) and insulin resistance (IR) in the general population. As the fact that NAFLD is associated with CHD and IR has been widely recognized, exploration on the relationship between serum remnant-C levels and NAFLD developing is also sorely needed.

In this longitudinal retrospective cohort study, we aimed to explore the association of the components of dyslipidemia and serum remnant-C levels with the occurrence of NAFLD in a Chinese cohort of middle aged and elderly individuals(middle-aged, 45~59 years old; elderly, 60 years old and above; the average age is 56.94 years; mainly from Shandong Province), in an attempt to expand our understanding of the remnant-C as a possible risk factor of NAFLD. To our knowledge, our study is the first and largest analysis specifically led to evaluate the association between serum remnant-C levels and NAFLD risk in a longitudinal retrospective cohort. Knowledge of this association is important for perfect health care resource allocation and prevention and management of NAFLD-related diseases, in turn to attenuate the society medical burden.

## 2 Methods

### 2.1 Ethical approval

The Ethics Committee of Shanghai Jiao Tong University and Shandong Provincial Hospital (NO.2021-323) approved this study which was conducted with the principles in the Declaration of Helsinki. And the consent obtained from all subjects included in the study was both informed and written.

### 2.2 Subjects

This is a longitudinal study and the data were retrospectively reviewed. All subjects in the current study are from the 13,876 individuals who recruited in the Shandong cohort of the REACTION study, which is a prospective, multicenter, observational cohort study conducted from 2011 and has recruited more than 200, 000 people in China so far (8). We included 6,634 participants who had more than one visit during the study period with an average follow-up time of 43.34 months and assessed for eligibility.

**Figure 1:**
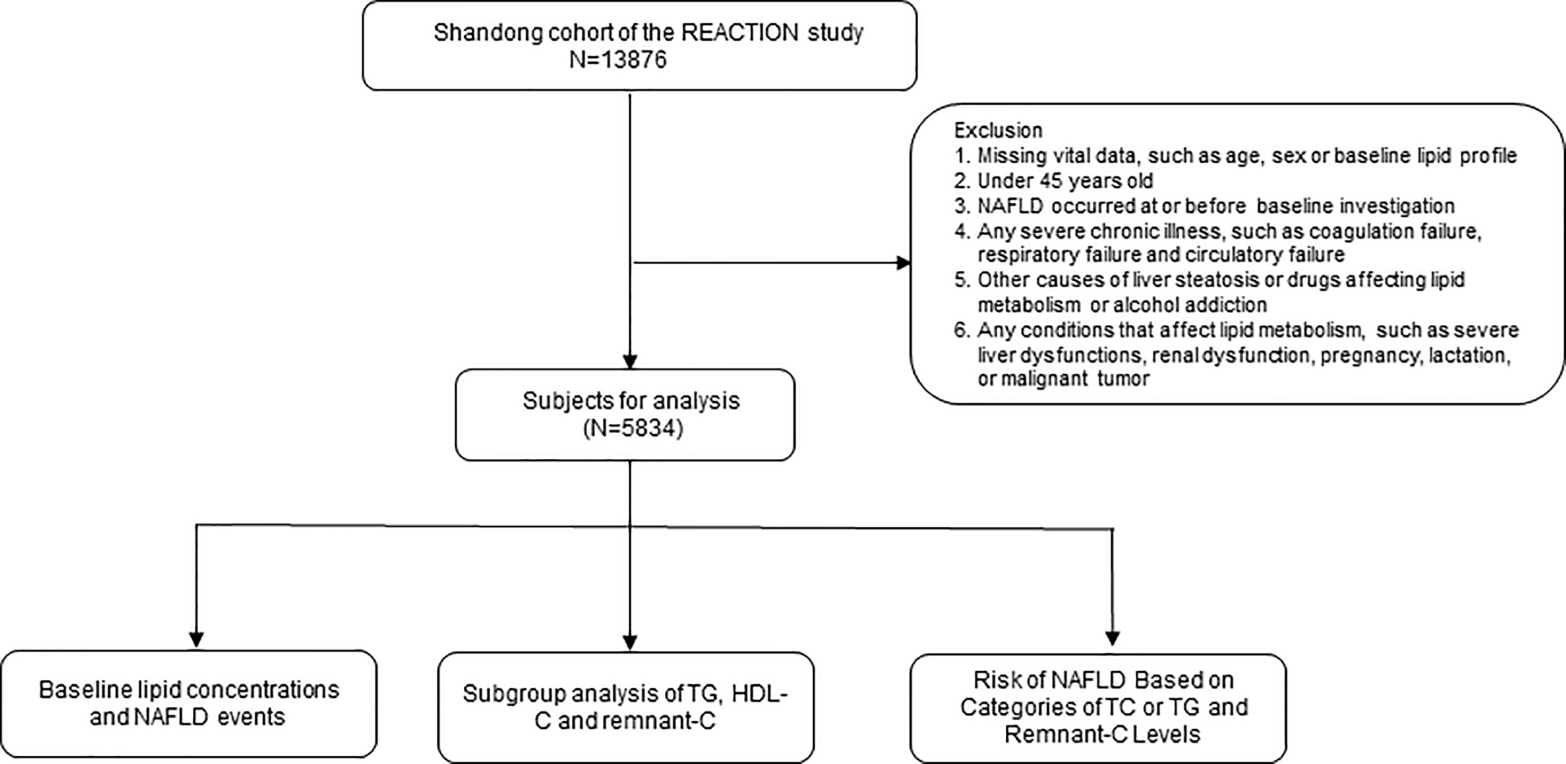
The flow chart of our study.

As our main research objective was to assess the association of TG and remnant-C with the outcome of NAFLD, for the present analysis we have excluded individuals with (1) missing vital data, such as age, sex, body mass index (BMI), or baseline lipid profile (2);under 45 years old (3); NAFLD has occurred at or before the baseline investigation; (4) any severe chronic illness, such as coagulation failure, respiratory failure and circulatory failure; (5) other causes of liver steatosis or drugs affecting lipid metabolism or alcohol addiction; and (6) any conditions that affect lipid metabolism, such as severe liver dysfunctions, renal dysfunction, pregnancy, lactation, or malignant tumor (**Figure 1**). The date of the first recruited participant was April 2011, and the end of the follow-up was July 2017.

### 2.3 Measurements

Data was collected at local health stations by trained investigators to minimize instructor variability. Demographic characteristics were obtained from a well-established questionnaire through a face-to-face interview. All subjects were asked to fast for at least 10 hours before the health examinations. When subjects wore light clothing and took off their shoes, height and weight were measured in kilograms and centimeters, respectively. BMI (kg/m^2^) was calculated by dividing weight by the square of the height. Waist circumference (WC) was measured at the midpoint between the lower rib cage and the iliac crest in centimeters. Hip circumference (HC) was measured at the widest protrusion of buttocks in centimeters. Using an electronic sphygmomanometer (HEM-7117; Omron, Kyoto, Japan), blood pressure was measured three times after 5 min rest, and the average of the three measurements was calculated.

Blood samples were collected in the morning after a minimum 10-hour fasting. Fasting plasma glucose (FPG) was measured within 2 hours using the glucose oxidase method. Using the VARIANT II Hemoglobin Testing System (Bio-Rad Laboratories), glycated hemoglobin (HbA1c) was measured by high-performance liquid chromatography. After serum and plasma samples were separated and then shipped by air to the Clinical Laboratory for Endocrinology, Shanghai Institute of Endocrine and Metabolic Diseases, the lipid profile measurements including TG, TC, LDL-C, and HDL-C were performed using an autoanalyzer (ADVIA-1650 Chemistry System, Bayer, Leverkusen, Germany). Remnant-C was estimated as TC minus LDL-C minus HDL-C.

Diabetes was defined as fasting blood glucose ≥7.0 mmol/L and/or HbA1c ≥6.2% and/or taking glucose- lowering medication and/or self- report of diabetes (9). The CHD definition and diagnostic criteria are included in the previous guidelines (10). In this study, CHD mainly refers to diagnosed angina pectoris, myocardial infarction, heart failure or coronary heart disease.

### 2.4 Outcome ascertainment

The primary outcome was NAFLD status at the end of the follow-up. The sources of information to identify outcome were yearly revisions of medical records by trained investigators and clinical technicians. All medical records related to outcome were evaluated by the outcome adjudication committee. As described by the Chinese Liver Disease Association, NAFLD was diagnosed by ultrasound (US) (2). In brief, the definition of NAFLD was a diffusion-enhanced near-field echo in the liver region and gradual decay of the far-field echo with one of the following conditions: mild to moderate hepatomegaly with peripheral and marginal passivation; the structure of the hepatic lacunae cannot clearly displayed; the blood flow distribution was normal, but the blood flow signal was reduced; or the unclear or incomplete right liver lobe and diaphragm muscle capsule (11).

### 2.5 Statistical analysis

The Kolmogorov-Smirnov Test was used to test the normality of all variables prior to performing parametric tests. Normally distributed continuous parameters were represented as the mean ± SD, while nonnormally distributed continuous variables were represented as the medians with interquartile ranges. Categorical variables were summarized as numbers (percentage). To analyze differences of remnant-C distribution at baseline between the sex, diabetes status, CHD status and BMI categories groups, data were tested by the nonparametric test.

Follow-up time was calculated as the interval between the date of recruitment in the study and the date of the incident NAFLD, the date of the last visit, or the last record of the deceased subjects while he or she was alive. The association between lipid concentrations (either as continuous or categorical variables) and incident NAFLD were evaluated by unadjusted and adjusted Cox proportional hazard models. The potential confounders that may affect the association between lipid concentrations and incident NAFLD were all adjusted in the Cox proportional hazard models, including age, sex, HC, BMI, systolic blood pressure (SBP), diastolic blood pressure (DBP), FPG, diabetes status and CHD status (12, 13). All *p* values were two-tailed, and *p* values less than 0.05 were considered statistically significant. SPSS version 25.0 (SPSS, Chicago, IL, USA) and R (version 4.1.1) was used for all parametric tests.

## 3 Results

### 3.1 Description of study subjects

**Table 1** lists the baseline characteristics of the subjects (N=5,834) in the current study. The participants’ average age was 56.94 years, 39.06% were men, median BMI was 25.08 kg/m^2^, median HC was 97cm. Diabetes and CHD were present in 29.16% and 6.05% of participants, respectively.

**Table 1:** Description of study subjects.

	Total	Female	Male	*p*
N	5834	3555	2279	
Age, yrs	56.94 ± 7.38	56.43 ± 7.24	57.74 ± 7.51	<0.001
HC, cm	97.00 (91.00,102.50)	97.00 (91.00,102.00)	97.00 (92.00,103.00)	0.086
BMI, kg/m^2^	25.08 (22.76,27.46)	25.20 (22.81,27.59)	24.96 (22.64,27.34)	0.024
SBP, mmHg	138 (125,153)	136 (123,152)	141 (128,155)	<0.001
DBP, mmHg	81 (74,89)	79 (72,87)	84 (76,92)	<0.001
FPG, mmol/L	5.83 (5.40,6.50)	5.77 (5.36,6.39)	5.94 (5.49,6.65)	<0.001
Diabetes	1701 (29.16)	988 (27.79)	713 (31.29)	0.004
CHD	353 (6.05)	219 (6.16)	134 (5.88)	0.661

Values are mean ± SD or median (IQR: interquartile range) and n. (%). HC, Hip circumference; BMI, Body mass index; SBP, Systolic blood pressure; DBP, Diastolic blood pressure; FPG, Fasting plasma glucose; CHD, Coronary heart disease.

### 3.2 Baseline lipid profile of study subjects

**Table 2** depicts the lipid profile at baseline of study subjects. Median TC and TG was 5.12 and 1.19 mmol/L, respectively. The lipid alterations characteristic of atherogenic dyslipidemia (TG>1.69 mmol/L and HDL-C<1.03 mmol/L in men or<1.29 mmol/L in women) are present in 9.65% of the baseline population.

**Table 2:** Baseline lipid profile of study subjects.

	All Participants	Female	Male	*p*
TC, mmol/L	5.12 (4.41,5.88)	5.20 (4.48,5.98)	5.00 (4.29,5.75)	<0.001
TG, mmol/L	1.19 (0.86,1.72)	1.18 (0.86,1.73)	1.19 (0.85,1.70)	0.801
HDL-C, mmol/L	1.40 (1.19,1.64)	1.43 (1.22,1.67)	1.34 (1.15,1.60)	<0.001
LDL-C, mmol/L	3.00 (2.44,3.63)	3.02 (2.47,3.67)	2.96 (2.4,3.57)	<0.001
TG/HDL	0.86 (0.57,1.35)	0.83 (0.57,1.33)	0.90 (0.58,1.39)	0.001
TG>1.69 mmol/l +HDL-C<1.03/1.29 mmol/L (in men/women)	563 (9.65)	454(12.77)	109 (4.78)	<0.001

Values are median (IQR: interquartile range) or n (%). TC, Total cholesterol; TG, Triglycerides; HDL-C, High-density lipoprotein cholesterol; LDL-C, Low-density lipoprotein cholesterol.

**Figure 2:**
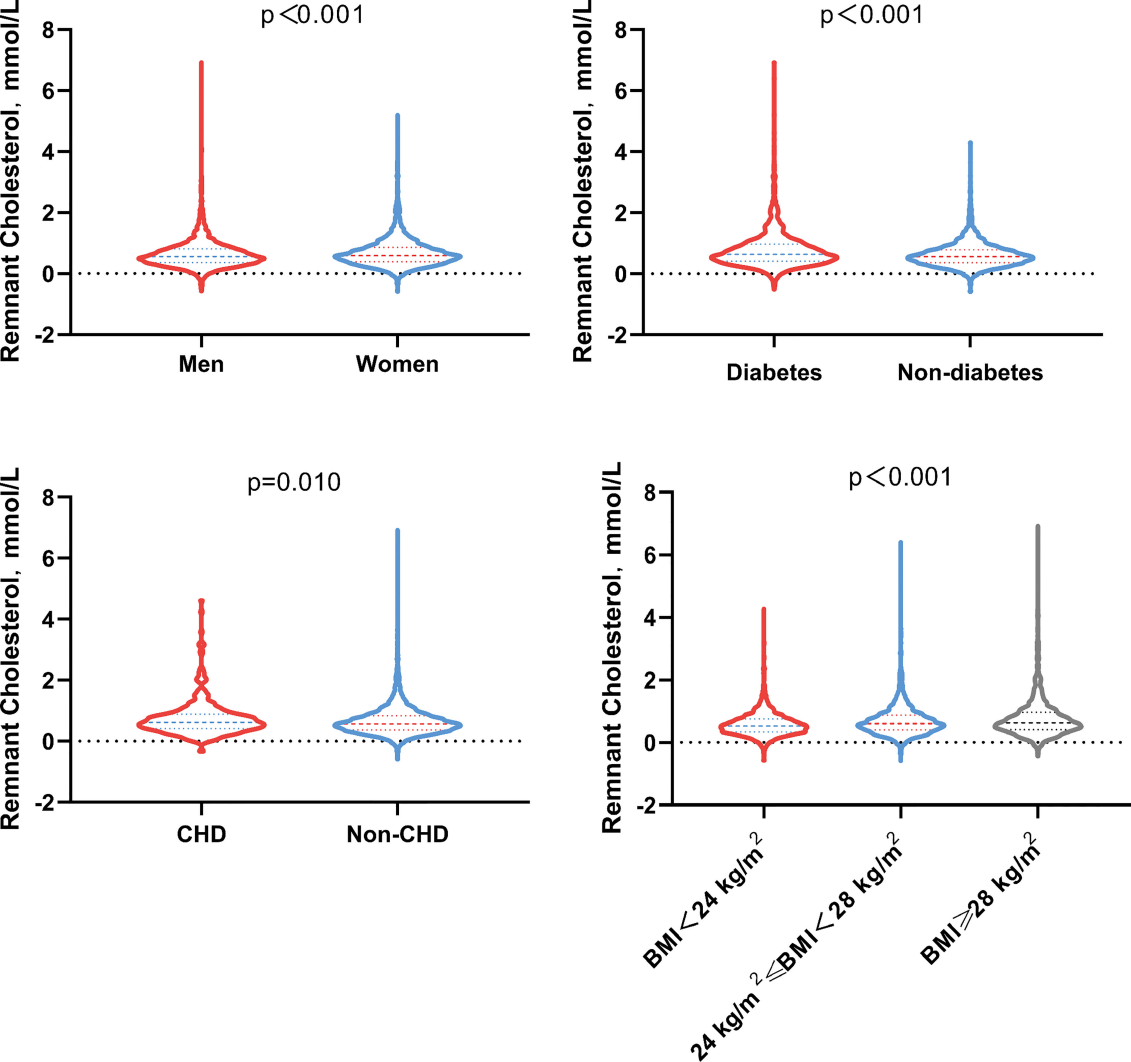
Remnant cholesterol distribution at baseline by sex, diabetes tatus, CHD status and BMI (kg/m^2^) categories. BMI, Body mass index; CHD, Coronary heart disease.

Median remnant-C was 0.58 mmol/L (**Table 3**), and its distribution was differed by BMI categories (<24 kg/m^2^:0.53(0.34,0.75) mmol/L; 24 to 28 kg/m^2^: 0.60(0.40,0.88)mmol/L; ≥28 kg/m^2^: 0.63(0.41,0.96) mmol/L), CHD status (no CHD: 0.57(0.37,0.84) mmol/L; CHD:0.62(0.41,0.89) mmol/L) and diabetes status (no diabetes:0.56(0.36,0.79) mmol/L; diabetes:0.64(0.41,0.97) mmol/L) (**Figure 2**).

**Table 3:** Baseline remnant-C and categories of TC and remnant-C levels of study subjects.

	All Participants	Female	Male	*p*
Remnant-C, mmol/L	0.58 (0.38,0.84)	0.59 (0.39,0.86)	0.56 (0.36,0.81)	<0.001
**TC and remnant-C groups**				<0.001
TC≤ 6.2mmol/L& remnant-C≤ 0.58mmol/L	2696 (46.21)	1561 (43.91)	1135 (49.80)	
TC≤ 6.2mmol/L& remnant-C>0.58mmol/L	2146 (36.78)	1309 (36.82)	837 (36.73)	
TC>6.2mmol/L& remnant-C≤ 0.58mmol/L	287 (4.92)	196 (5.51)	91 (3.99)	
TC>6.2mmol/L& remnant-C>0.58mmol/L	705 (12.08)	489 (13.76)	216 (9.48)	
**TG and remnant-C groups**				0.004
TG≤ 1.7mmol/L& remnant-C≤ 0.58mmol/L	2833 (48.56)	1665 (46.84)	1168 (51.25)	
TG≤ 1.7mmol/L& remnant-C>0.58mmol/L	1524 (26.12)	981 (27.59)	543 (23.83)	
TG>1.7mmol/L& remnant-C≤ 0.58mmol/L	150 (2.57)	92 (2.59)	58 (2.54)	
TG>1.7mmol/L& remnant-C>0.58mmol/L	1327 (22.75)	817 (22.98)	510 (22.38)	

Values are median (IQR: interquartile range) or n (%). Remnant-C, Remnant-cholesterol; TC, Total cholesterol; TG, Triglycerides.

### 3.3 Baseline lipid concentrations and NAFLD events

After adjusting for age, sex, HC, BMI, SBP, DBP, FPG, CHD and diabetes, the serum concentrations of TG were associated with an 8.0% higher risk of NAFLD per every 1-SD increase, whereas serum remnant-C was associated with a 14.3% higher risk per every 1-SD increase (**Table 4**). Conversely, for every 1-SD increase in HDL-C, the risk of NAFLD decreased 42.9% (HR = 0.571, 95%CI:0.487-0.670, *p*< 0.001). The lipid alterations characteristic of atherogenic dyslipidemia (TG>1.69 mmol/L and HDL-C<1.03 mmol/L in men or<1.29 mmol/L in women) were also associated with a 34.3% higher risk of the incident NAFLD. No significant interactions were observed between TC (*p*=0.371) or LDL-C (*p*=0.597) and the risk of NAFLD.

**Table 4:** Association of baseline lipid values with NAFLD outcomes.

	No Event (n=4,165)	Event (n=1,669)	Hazard Ratio (95% CI)	*p*
TG, mmol/L	1.10 (0.81,1.57)	1.43 (1.01,2.16)	1.080 (1.047-1.113)	<0.001
HDL-C, mmol/L	1.43 (1.22,1.69)	1.33 (1.12,1.53)	0.571 (0.487-0.670)	<0.001
LDL-C, mmol/L	2.98 (2.43,3.59)	3.06 (2.49,3.72)	0.985 (0.931-1.042)	0.597
TC, mmol/L	5.10 (4.40,5.84)	5.21 (4.45,5.98)	0.980 (0.938- 1.024)	0.371
Remnant-C, mmol/L	0.56 (0.36,0.80)	0.63 (0.42,0.94)	1.143 (1.052-1.242)	0.002
TG**/**HDL	0.77 (0.52,1.19)	1.07 (0.73,1.76)	1.072 (1.042,1.103)	<0.001
TG>1.69 mmol/L +HDL-C< 1.03/1.29 mmol/L (in men/women)	262 (6.29)	301 (18.03)	1.343 (1.177-1.533)	<0.001

Values are median (IQR: interquartile range) or n (%), unless otherwise indicated. Hazard ratios (HRs) were estimated by Cox proportional hazards regression models adjusted for age, sex, hip circumference, body mass index, systolic blood pressure, diastolic blood pressure, fasting plasma glucose, coronary heart disease and diabetes. The “TG”, “HDL-C”, “LDL-C”, “TC”, “Remnant-C”, “TG/HDL” and the “TG>1.69 mmol/L +HDL-C< 1.03/1.29 mmol/L (in men/women)” are included in the Cox model, respectively. CI, confidence interval; other abbreviations as in **Table 2**.

### 3.4 Subgroup analysis of TG, HDL-C and remnant-C

**Figure 3:**
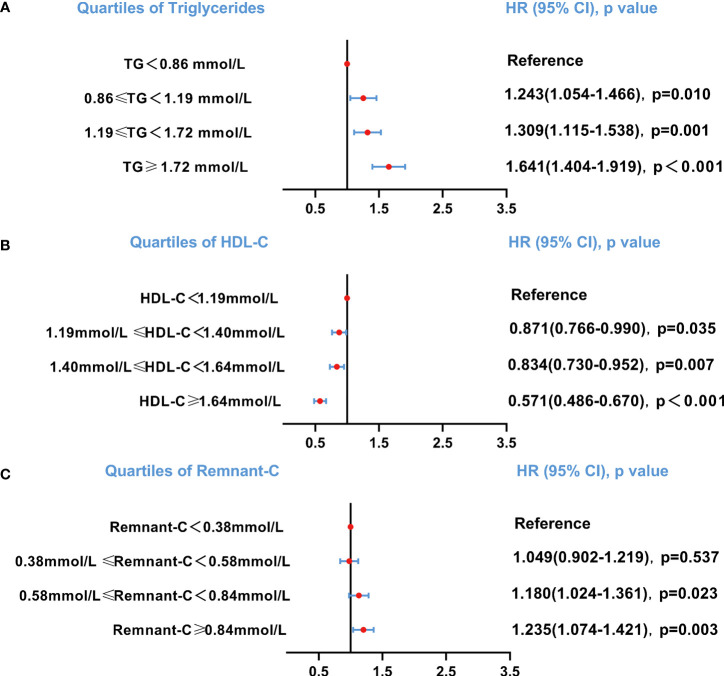
Risk of NAFLD across quartiles of baseline lipid parameters. To assess the risk of Non-alcoholic fatty liver disease (NAFLD) associated with the baseline lipid values of interest, we calculated hazard ratios (HR) for the second, third, and fourth quartiles (compared with the first quartile) of levels of **(A)** Triglycerides (TG), **(B)** High-density lipoprotein cholesterol (HDL-c), and**(C)** Remnant cholesterol (Remnant-C). Analyses were adjusted for age, sex, hip circumference, body mass index, systolic blood pressure, diastolic blood pressure, fasting plasma glucose, cardiovascular disease and diabetes. CI, confidence interval.

In order to better understand the risk factors in the lipid profile that may affect NAFLD incidence and to further identify potential information, subgroup analysis of TG, HDL-C and remnant-C was performed. As the serum concentrations of TG increase, the risk of the incident NAFLD increases. On the contrary, the risk of NAFLD decreases as the HDL-C levels increase. Particularly, the incidence of NAFLD was high in subjects in the upper quartiles of the remnant-C compared with the lowest quartiles (HR = 1.235, 95%CI:1.074-1.421, *p*=0.003) (**Figure 3**).

### 3.5 Risk of NAFLD based on categories of TC or TG and remnant-C levels

**Figure 4:**
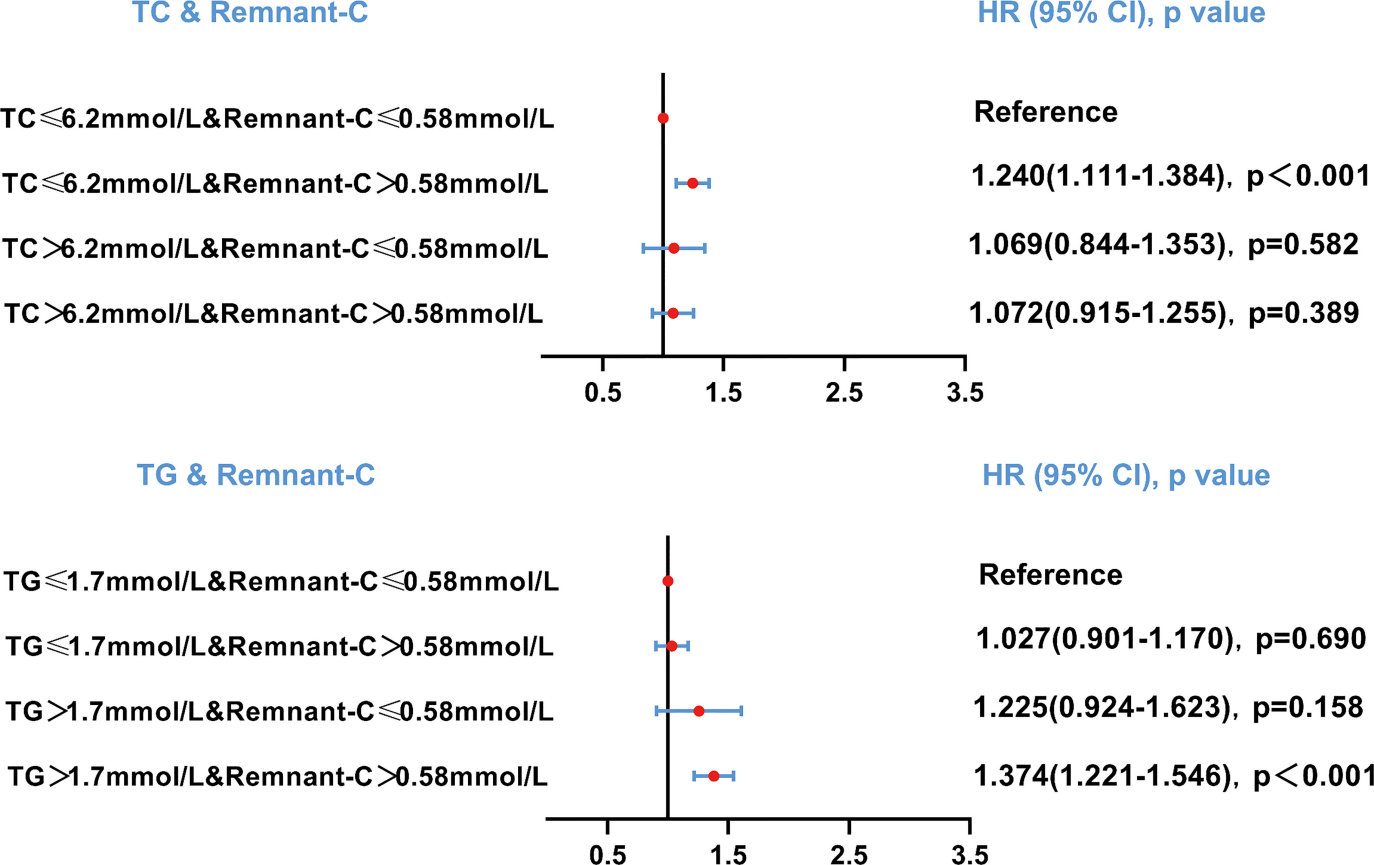
Risk of NAFLD based on categories of TC or TG and remnant-C levels. Data were adjusted for age, sex, hip circumference, body mass index, systolic blood pressure, diastolic blood pressure, fasting plasma glucose, cardiovascular disease and diabetes. HR, hazard ratio; CI, confidence interval; Remnant-C, Remnant-cholesterol; TC, Total cholesterol; TG, Triglycerides.

**Figure 5:**
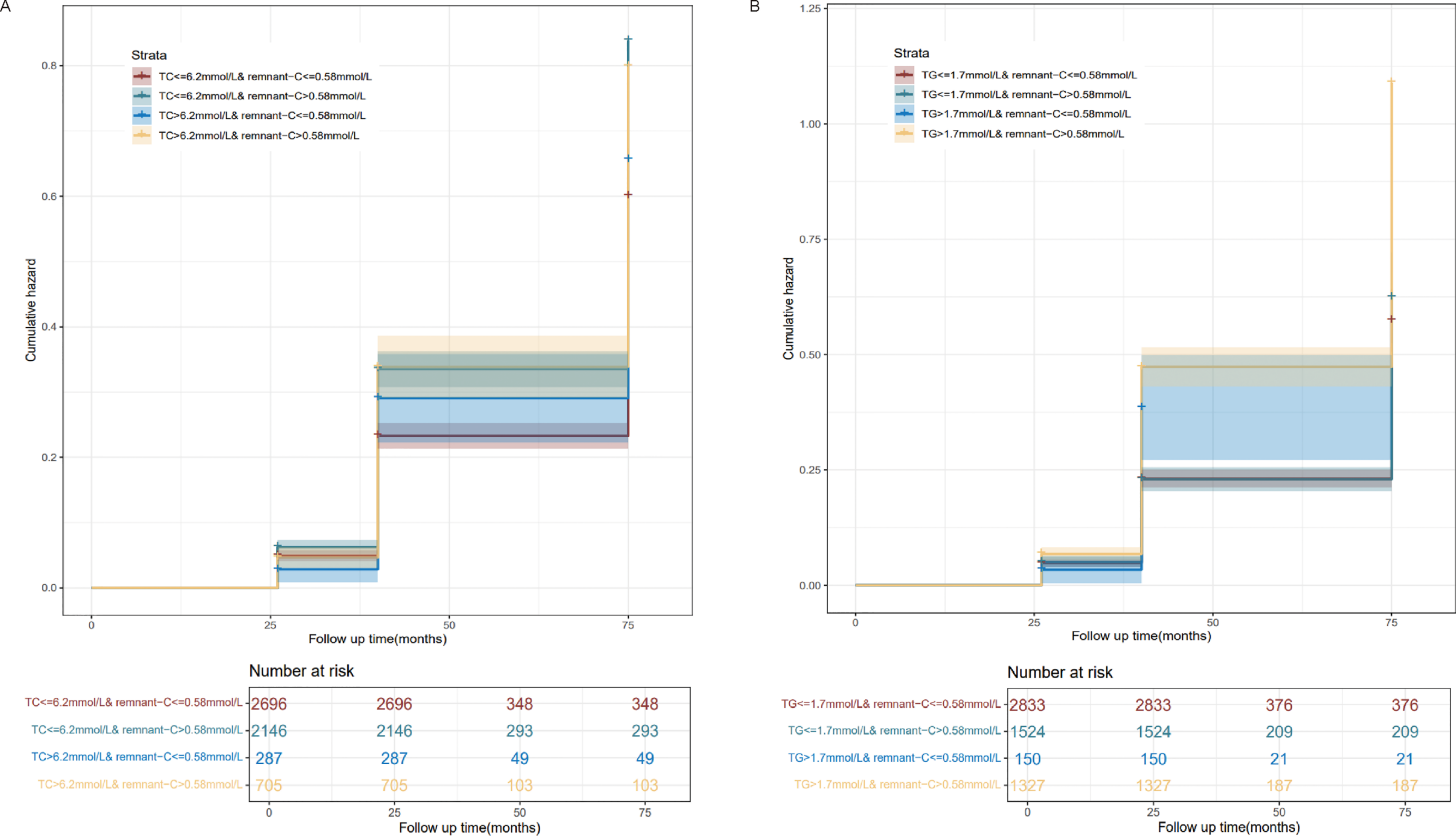
Incidence curves of NAFLD based on pre-defined categories of TC or TG and remnant-C levels. **(A)** Pre-defined categories of TC and remnant-C levels. **(B)** Pre-defined categories of TG and remnant-C levels. Remnant-C, Remnant-cholesterol; TC, Total cholesterol; TG, Triglycerides.

As the residues rich in cholesterol esters and one of the components of serum TC, the relationship between the different combinations of remnant-C and TC levels and the occurrence of NAFLD is worth exploring. The abnormally high levels of remnant-C were defined to the remnant-C>50th percentile of the cohort (0.58mmol/L). Conventionally, high levels for TC were defined as >6.2mmol/L. When TC values ≤ 6.2mmol/L, high baseline remnant-C identified subjects are at a higher risk of NAFLD compared with those at lower concentrations (**Figure 4**). When TG values > 1.7mmol/L, high baseline remnant-C identified subjects are at a higher risk of NAFLD compared with those at lower concentrations (**Figure 4**). Data were adjusted for age, sex, HC, BMI, SBP, DBP, FPG, CHD and diabetes. Cumulative hazard curve was constructed to assess the incidence of NAFLD by categories of low and high TC or TG and remnant-C. NAFLD incidence was low in the low remnant-C groups, regardless of TC levels (**Figure 5**).

### 3.6 Sensitivity analysis

The distributions of remnant-C were differed by sex groups, BMI categories, cardiovascular disease status and diabetes status. Next, we supplemented some subgroup analyses for these factors to explore the robust associations of remnant-C or other lipids with NAFLD. After adjusting for other factors in the Cox regression models, we found that serum levels of TG and remnant-C, but not TC or LDL-C, were associated with NAFLD outcomes in women group (**Supplementary Figure 1**), non-cardiovascular disease status (**Supplementary Figure 2**), non-diabetes status (**Supplementary Figure 3**) and middle BMI categories (24 to 28 kg/m^2^) (**Supplementary Figure 4**).

## 4 Discussion

Among the middle aged and elderly subset of the Chinese population in this longitudinal retrospective cohort study, the main findings were that serum levels of TG and remnant-C, but not TC or LDL-C, were associated with NAFLD outcomes, independent of other risk factors, regardless of age, sex, HC, BMI, SBP, DBP, FPG, CHD and diabetes. To the best of our knowledge, this is the first epidemiological and longitudinal study reporting the association between serum remnant-C and the risk of NAFLD outcomes.

Methods to assess risk factors for NAFLD may include autopsy studies, hospital population-based studies, and community population-based screening studies. Autopsy can easily provide evidence of NAFLD, but the main limitation of autopsy research is that it cannot provide the true prevalence of NAFLD in living people. Because NAFLD patients may be asymptomatic and often do not go to the hospital, studies based on the hospital population cannot provide true risk factors for NAFLD. In contrast, a community-based population-based screening study of random samples can more objectively assess the true risk factors of NAFLD. In the diagnosis of NAFLD, US has a sensitivity of 80%-95% and a specificity of 90%-95% and has a wide range of application value in the screening of NAFLD in the general population (14–16). In addition, reports from Japan showed that NAFLD was more prevalent in the middle-aged subjects (17). Hence, using a community US survey, we determined the risk factors of NAFLD in a Chinese cohort of middle aged and elderly individuals.

Our longitudinal cohort results confirmed previous cross-sectional evidence on the risk role of TG in NAFLD, that is, the increase concentrations of serum TG were associated with higher risk of NAFLD as time goes by. In the characterization of the pathogenic mechanisms of NAFLD, the ‘first hit’ is triggered by the lipid accumulation in hepatocytes, a trait in which exacerbated fat intake and IR would play a key role (18). Although NAFLD is associated with excess triglycerides in the liver, current evidence suggests that free fatty acids (FFAs), not TG, accumulate in lipid droplets to cause inflammatory liver damage in nonalcoholic steatohepatitis. The liver metabolism of FFAs leads to the formation of toxic metabolites, which are mainly responsible for the production of oxidative stress, inflammation and liver parenchymal damage (19–21). However, the accumulation of TG in the liver is currently considered to be a non-toxic and safer form of liver lipid storage, which is an epiphenomenon that reflects changes in the balance of hepatocyte FFA flux and cellular stress (22); therefore, steatosis can be recognized as an early adaptive response to hepatocyte stress as a result of increased caloric consumption. Through this process, potentially lipotoxic FFAs are segmented into relatively inert intracellular TG molecules (23). Many studies have found that IR is the most important and common potential factor involved in the accumulation of free fatty acids in the liver. The current dominant paradigm is that IR leads to dyslipidemia. Our results show that serum TG concentration is a risk factor for NAFLD over time. So, is hypertriglyceridemia triggering IR or IR causing changes in serum TG concentration in the occurrence of NAFLD? It is important to determine whether hypertriglyceridemia plays a causal role in the etiology of insulin resistance in NAFLD since it can reveal new avenues to combat NAFLD.

Remnant-C is the residue of the TRLs metabolism that consists of chylomicron remnants in the non-fasting state and VLDL and intermediate density lipoproteins in the fasting state. Previous evidence depicted remnant-C was associated with the increased risk of major adverse cardiovascular events (MACEs), but the relationship between serum remnant-C level and the occurrence of NAFLD has not been studied longitudinally (24). The study from Italian hospitals including 798 unselected patients with cardio-metabolic diseases and 79.2% with the presence of NAFLD showed that there was a correlation between values of the circulating remnant-C levels and liver disease severity in patients with NAFLD (25). Consistent with this, teenagers with high remnant-C levels had more severe fat accumulation in their livers compared to those with low remnant-C levels in the Raine Study (26).Particularly, in our longitudinal retrospective cohort study, we found a significant correlation between levels of serum remnant-C and the incidence of NAFLD after adjusting for age, sex, HC, BMI, SBP, DBP, FPG, CHD and diabetes. In other words, serum remnant-C was associated with a 14.3% higher risk per every 1-SD increase and the incidence of NAFLD was high in subjects in the upper quartiles of the remnant-C compared with the lowest quartiles (HR = 1.235, 95%CI:1.074-1.421, *p*=0.003), which can expand our knowledge of the remnant-C as a possible risk factor of NAFLD to some extent.

Atherogenic dyslipidemia, characterized by plasma hypertriglyceridemia, increased small dense LDL particles, and decreased serum HDL-C concentration, is often present in a wide range of chronic cardio-metabolic disorders within the NAFLD, overweight, obesity and diabetes and considered as one of the main causes of lipid-dependent residual risk, regardless of LDL-C concentration (27–29). It’s worth noting that we also found the lipid alterations characteristic of atherogenic dyslipidemia were associated with a 34.3% higher risk of the incident NAFLD. Aside from IR, several other factors include diet composition, gut microbiota and genetic factors also contribute to the pathogenesis of atherogenic dyslipidemia in patients with NAFLD (30).

Furthermore, remnant-C, but not TC, was the major cholesterol fraction contributor to NAFLD in our cohort of participants who had no previous NAFLD. No significant interactions were observed between TC and the risk of NAFLD. Cumulative hazard curve, constructed to assess the incidence of NAFLD by categories of low and high TC and remnant-C, found that NAFLD incidence was low in the low remnant-C groups, regardless of TC levels. It is critical to identify potentially modifiable risk factors of NAFLD is of importance, so as to help develop targeted therapies that decrease the risk of NAFLD. In addition, consistent with the study by Catanzaro R et al. (31), we demonstrated that higher TG/HDL-C ratio is associated with NAFLD, so TG/HDL-C could be used as a reliable non-invasive marker in diagnostics of NAFLD in the future. Notably, our subgroup analyses found that serum levels of TG and remnant-C, but not TC or LDL-C, were associated with NAFLD outcomes in women group, non-cardiovascular disease status, non-diabetes status and middle BMI categories (24 to 28 kg/m^2^). Therefore, more TG and remnant-C monitoring should be given to individuals with the above characteristics for early prevention and intervention in the occurrence and development of NAFLD.  

Recently, the relevant data of Guideline for the Management of Diabetes Mellitus in the Elderly in China (2021 edition) (32) show that the prevalence of diabetes in the elderly in China is 30.2%, which is much higher than the 19.3% of diabetes prevalence in the elderly in the world. The number of patients suffering from diabetes in China reached 35.5 million, accounting for a quarter of the world’s elderly diabetes and ranking first in the world. Our population fits this prevalence rate, so the results of the study are of great significance.

The study is not without its limitations. First, our research was observational and the causal role of remnant-C on the risk of NAFLD incident should be verified in further studies. Second, since we focused on plasma lipid related levels, we collected data on a patient’s BMI, blood pressure, and other relevant indicators. We failed to measure or collect data on diet and physical exercise, which are very important influencing factors for NAFLD. And the lack of data on plasma insulin that could drive dyslipidemia or NAFLD may influencing the presented data. Third, the value of remnant-C in our study might have been overestimated by indirect calculation in comparison to direct measurement and more complicated and expensive measurement of remnant-C could be required for accurate results in vulnerable patients.

## 5 Conclusions

In summary, our study identified levels of TG and remnant-C, but not TC or LDL-C, were associated with NAFLD outcomes independent of other risk factors in the middle aged and elderly subset of the Chinese population, especially in those who were women, non-cardiovascular disease status, non-diabetes status and middle BMI status (24 to 28 kg/m^2^). Consequently, the demonstration of an association between TG or remnant-C and NAFLD in those individuals could aid in the identification of subjects who might benefit from targeted risk factor assessment and management before the occurrence of adverse NAFLD outcomes.

## Data availability statement

The raw data supporting the conclusions of this article will be made available by the authors, without undue reservation. Requests for access should be directed to Chao Xu, at doctorxuchao@163.com.

## Author contributions

YC, CX and LG design the study. YC and QZ performed statistical analyses. FY, HL and GZ contributed to the critical revision of the manuscript. FY, PS and XZ contributed to the statical review. All authors contributed to the article and approved the submitted version.

## Supplementary material

Supplementary Figure 1 Sensitivity analysis for the associations of remnant-C or other lipids with incident NAFLD in sex groups. Data were adjusted for age, hip circumference, body mass index, systolic blood pressure, diastolic blood pressure, fasting plasma glucose, cardiovascular disease and diabetes status. HR, hazard ratio; CI, confidence interval; other abbreviations as in Table 2.

Supplementary Figure 2 Sensitivity analysis for the associations of remnant-C or other lipids with incident NAFLD in different diabetes status. Data were adjusted for age, sex, hip circumference, body mass index, systolic blood pressure, diastolic blood pressure, fasting plasma glucose and cardiovascular disease status. HR, hazard ratio; CI, confidence interval; other abbreviations as in Table 2.

Supplementary Figure 3 Sensitivity analysis for the associations of remnant-C or other lipids with incident NAFLD in different cardiovascular disease status. Data were adjusted for age, sex, hip circumference, body mass index, systolic blood pressure, diastolic blood pressure, fasting plasma glucose and diabetes status. HR, hazard ratio; CI, confidence interval; other abbreviations as in Table 2.

Supplementary Figure 4 Sensitivity analysis for the associations of remnant-C or other lipids with incident NAFLD in different body mass index categories. Data were adjusted for age, sex, hip circumference, body mass index, systolic blood pressure, diastolic blood pressure, fasting plasma glucose, cardiovascular disease and diabetes status. HR, hazard ratio; CI, confidence interval; BMI, body mass index; other abbreviations as in Table 2.
