# Supplementary figures and images for: Remnant cholesterol, stronger than triglycerides, is associated with incident non-alcoholic fatty liver disease
Source: Front Endocrinol (Lausanne). 2023 May 5;14:1098078. doi: 10.3389/fendo.2023.1098078 (PMC10198261; doi:10.3389/fendo.2023.1098078)

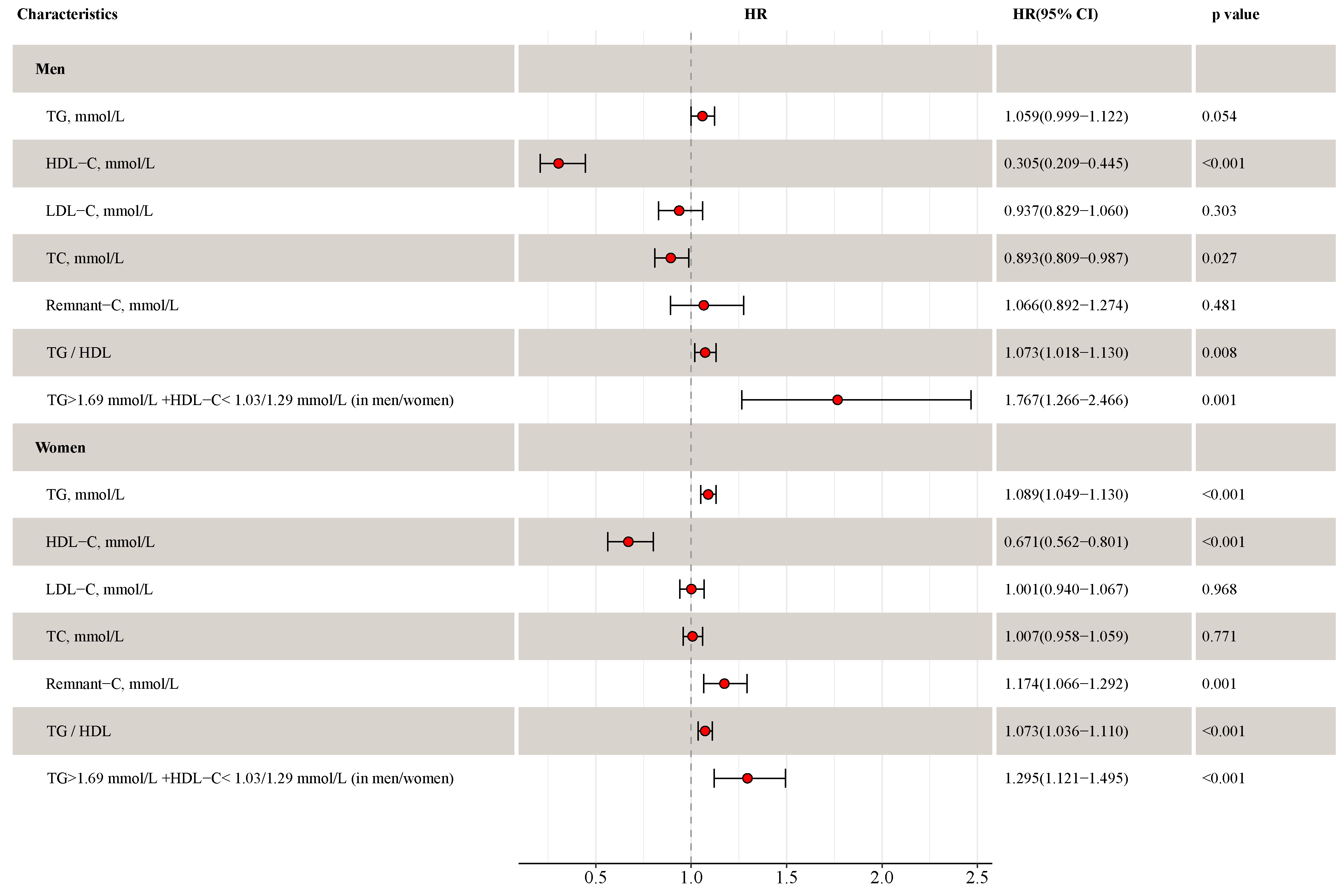

Supplement: Supplementary Figure 1 — Sensitivity analysis for the associations of remnant-C or other lipids with incident NAFLD in sex groups. Data were adjusted for age, hip circumference, body mass index, systolic blood pressure, diastolic blood pressure, fasting plasma glucose, cardiovascular disease and diabetes status. HR, hazard ratio; CI, confidence interval; other abbreviations as in Table 2. [file Image_1.tif]

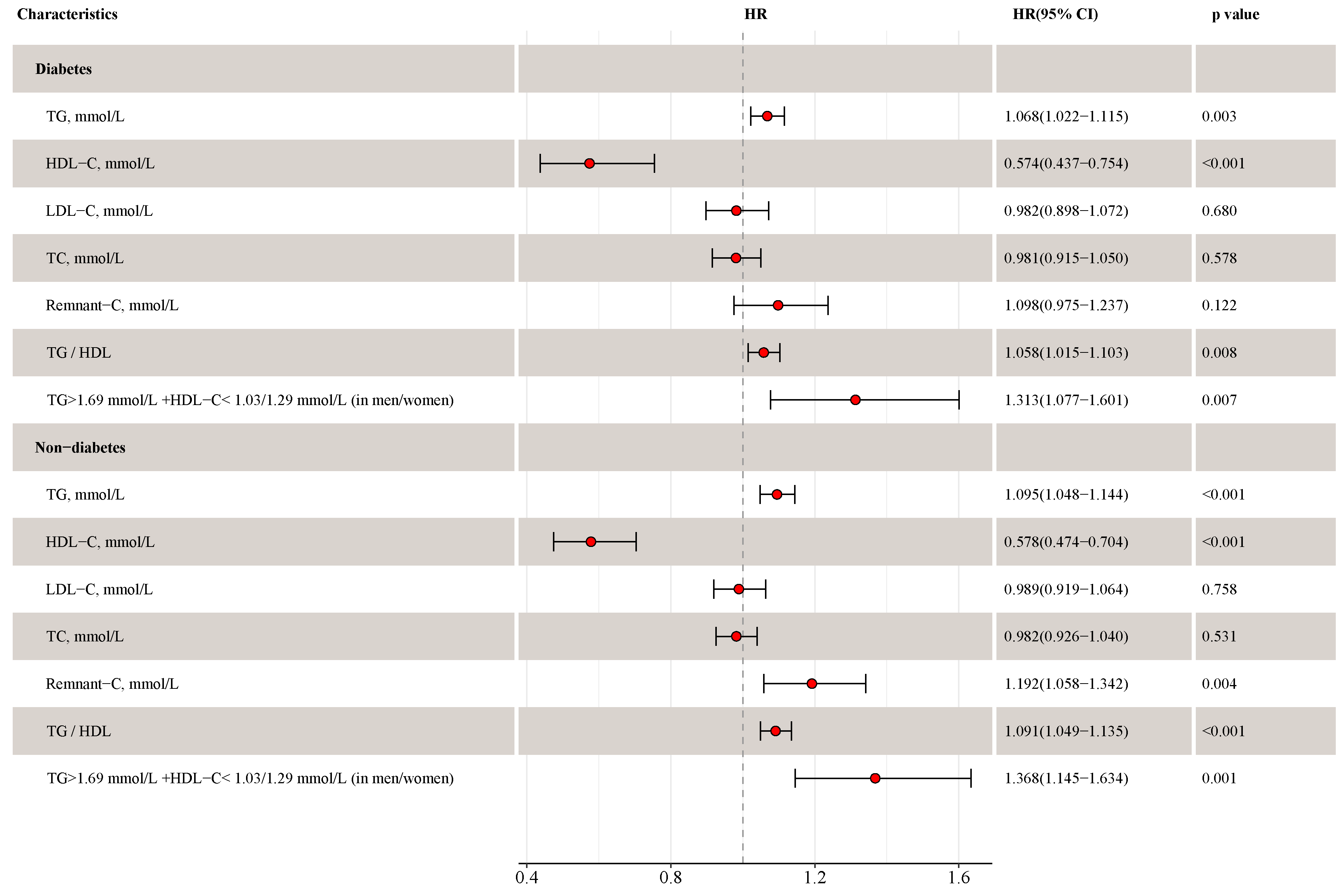

Supplement: Supplementary Figure 2 — Sensitivity analysis for the associations of remnant-C or other lipids with incident NAFLD in different diabetes status. Data were adjusted for age, sex, hip circumference, body mass index, systolic blood pressure, diastolic blood pressure, fasting plasma glucose and cardiovascular disease status. HR, hazard ratio; CI, confidence interval; other abbreviations as in Table 2. [file Image_2.tif]

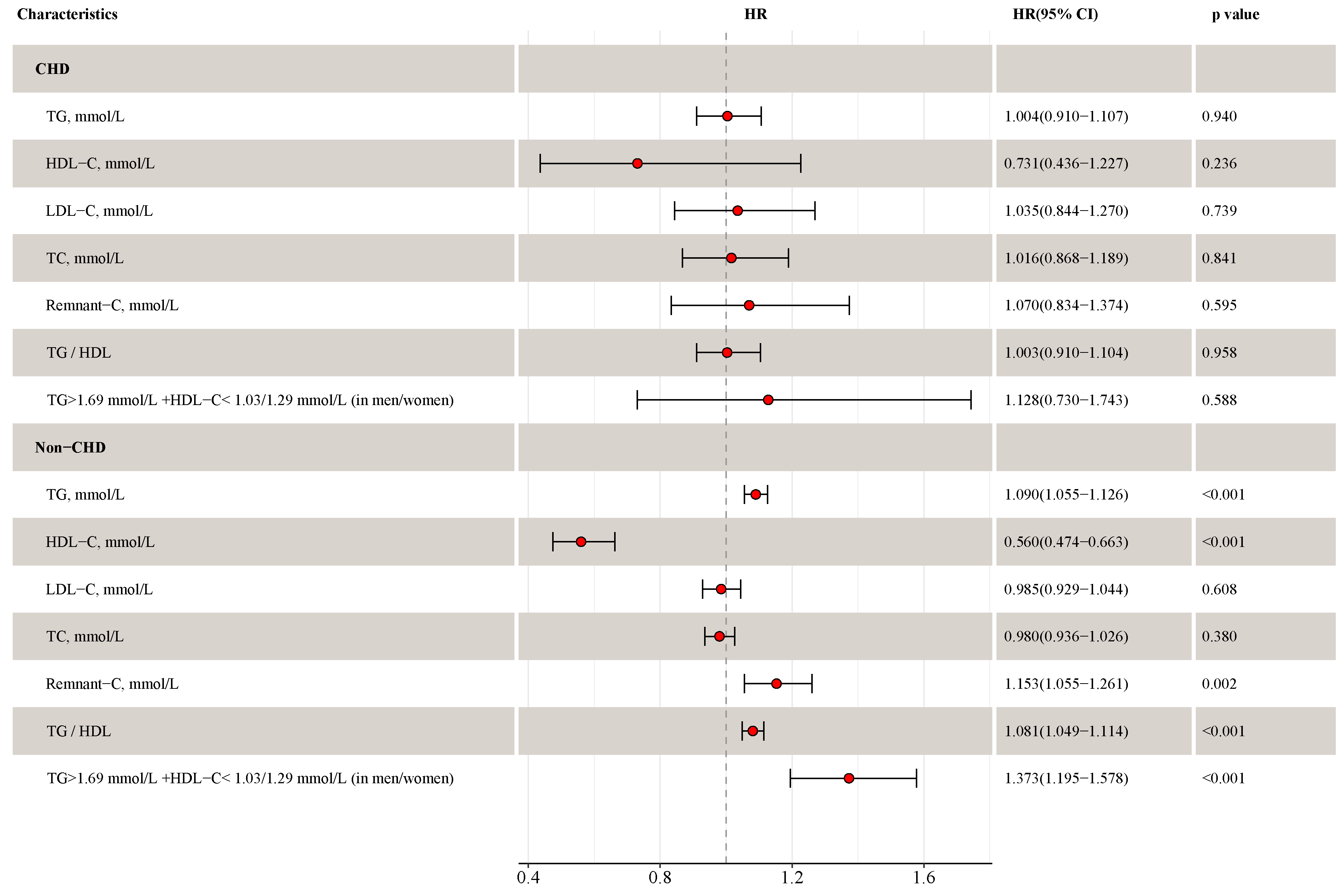

Supplement: Supplementary Figure 3 — Sensitivity analysis for the associations of remnant-C or other lipids with incident NAFLD in different cardiovascular disease status. Data were adjusted for age, sex, hip circumference, body mass index, systolic blood pressure, diastolic blood pressure, fasting plasma glucose and diabetes status. HR, hazard ratio; CI, confidence interval; other abbreviations as in Table 2. [file Image_3.tif]

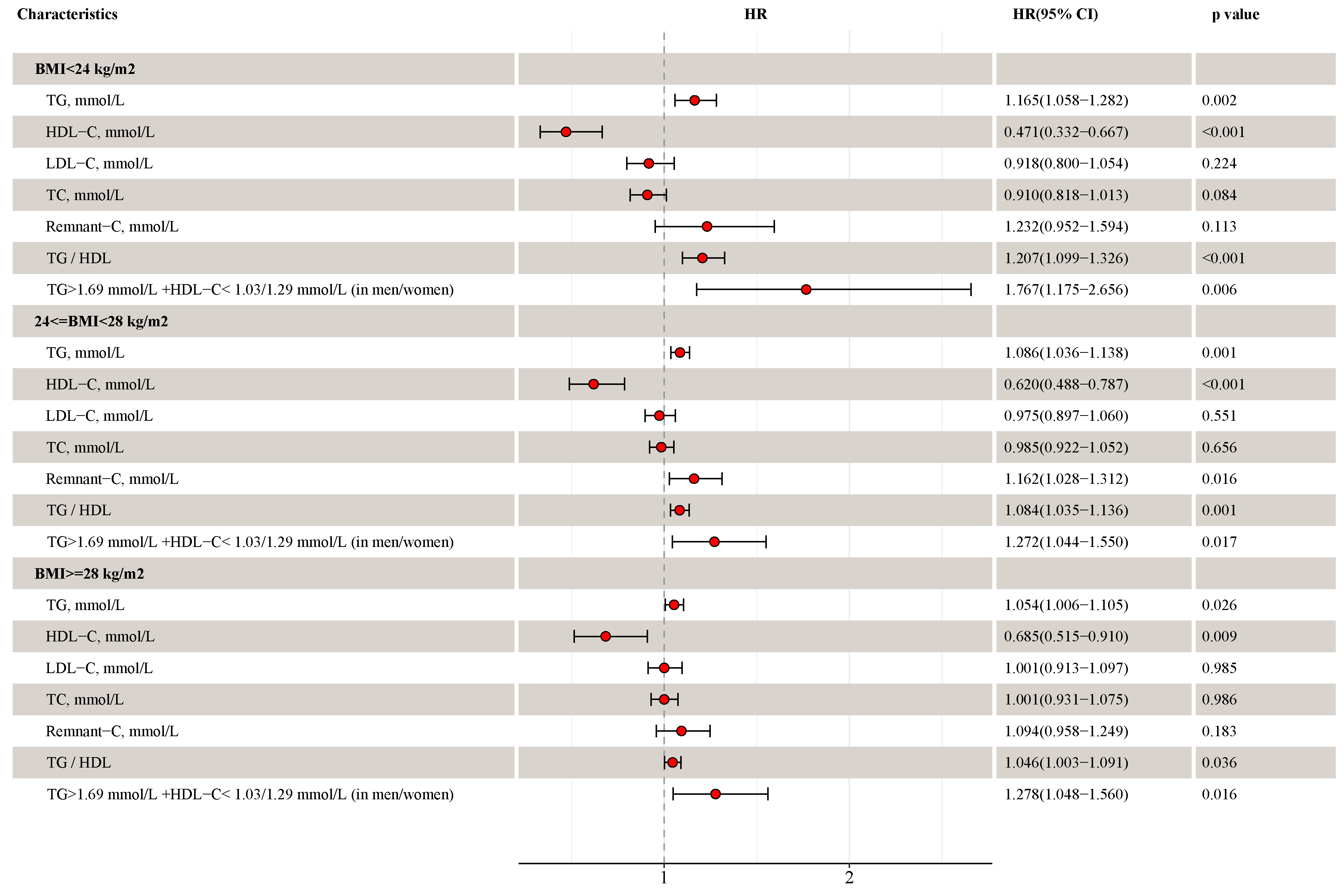

Supplement: Supplementary Figure 4 — Sensitivity analysis for the associations of remnant-C or other lipids with incident NAFLD in different body mass index categories. Data were adjusted for age, sex, hip circumference, body mass index, systolic blood pressure, diastolic blood pressure, fasting plasma glucose, cardiovascular disease and diabetes status. HR, hazard ratio; CI, confidence interval; BMI, body mass index; other abbreviations as in Table 2. [file Image_4.tif]
